# Metabolic differences and differentially expressed genes between C57BL/6J and C57BL/6N mice substrains

**DOI:** 10.1371/journal.pone.0271651

**Published:** 2022-12-22

**Authors:** Shino Nemoto, Tetsuya Kubota, Hiroshi Ohno

**Affiliations:** 1 Laboratory for Intestinal Ecosystem, RIKEN Center for Integrative Medical Sciences, Kanagawa, Japan; 2 Division of Diabetes and Metabolism, The Institute of Medical Science, Asahi Life Foundation, Tokyo, Japan; 3 Department of Clinical Nutrition, National Institutes of Biomedical Innovation, Health and Nutrition (NIBIOHN), Tokyo, Japan; 4 Division of Cardiovascular Medicine, Toho University Ohashi Medical Center, Tokyo, Japan; 5 Department of Diabetes and Metabolic Diseases, Graduate School of Medicine, The University of Tokyo, Tokyo, Japan; 6 Laboratory for Immune Regulation, Graduate School of Medical and Pharmaceutical Sciences, Chiba University, Chiba, Japan; 7 Immunobiology Laboratory, Graduate School of Medical Life Science, Yokohama City University, Yokohama, Japan; Oregon Health and Science University, UNITED STATES

## Abstract

C57BL/6J (B6J) and C57BL/6N (B6N) mice are the most frequently used substrains in C57BL/6 (B6) inbred mice, serving as physiological models for *in vivo* studies and as background strains to build transgenic mice. However, the differences in metabolic phenotypes between B6J and B6N mice are not coherent, and genotypic differences in metabolically important tissues have not been well studied. The phenotypic differences between B6J and B6N substrains have often been attributed to the role of the nicotinamide nucleotide transhydrogenase (*Nnt*) gene, whereby B6J has a spontaneous missense mutation of *Nnt*. Nevertheless, phenotypic differences between the two cannot be explained by *Nnt* mutations alone, especially in metabolic traits. Therefore, we aimed to investigate the genetic cause of the phenotypic differences between B6J and B6N mice. Determining consistent genetic differences across multiple tissues involved in metabolic traits such as subcutaneous and visceral white adipose tissues, brown adipose tissue, skeletal muscle, liver, hypothalamus, and hippocampus, may help explain phenotypic differences in metabolism between the two substrains. We report candidate genes along with comparative data on body weight, tissue weight, blood components involved in metabolism, and energy balance of B6J and B6N mice. Insulin degrading enzyme, adenylosuccinate synthase 2, and ectonucleotide triphosphate diphosphohydrolase 4 were highly expressed in B6J mice compared with those in B6N mice, and *Nnt*, WD repeat and FYVE domain containing 1, and dynein light chain Tctex-type 1 were less expressed in B6J mice compared with those in B6N mice in all seven tissues. Considering the extremely wide use of both substrains and their critical importance in generating transgenic and knock-out models, these findings guide future research across several interrelated fields.

## Introduction

C57BL-derived inbred mouse B6 is currently the most frequently used laboratory animal and is a vital tool in various biomedical studies, with more than 20 different substrains. Among these substrains, B6J and B6N mice are the most commonly used, owing to their strain stability and ease of breeding. B6J was the first mouse substrain that had its genome fully sequenced [1]; thus, many transgenic mice, including those produced using the Cre-lox and FLP-FRT recombination systems, have been generated with a B6J background, and *in vivo* studies using B6J as physiological or pathological models have increased rapidly. However, the B6N substrain has recently become more common and standardized because it was used as the embryonic stem cell line for large-scale knockout generation and phenotyping projects (*e*.*g*., International Knockout Mouse Consortium, International Mouse Phenotyping Consortium, and NIH Knockout Mouse Project), generating more than 5,000 targeted mutant mouse lines that are currently available to researchers worldwide [2]. Thus, both the B6J and B6N mice substrains have become indispensable and exclusive materials. Nevertheless, researchers are indifferent to the genetic and phenotypic differences between the two substrains [3, 4], and generate mice with mixed substrains [5], or do not explicitly address the substrain distinction in their publications [6]. Moreover, these unaddressed differences can lead to confounding experimental outcomes or misinterpretations; therefore, comprehensive information about the properties of these mouse substrains needs to be ascertained.

To date, various genomic differences such as indels, structural variations, and single nucleotide polymorphisms (SNPs), as well as phenotypic differences between B6J and B6N, have been reviewed [3, 7], some of which are known to be associated with pathogenesis and require caution when conducting experiments in such areas. For instance, ophthalmic problems have been known to occur in B6N mice [7, 8], regardless of genetic engineering [9, 10], as the substrain carries a single nucleotide deletion that causes a frameshift mutation and subsequent protein truncation in the crumbs cell polarity complex component 1 (*Crb1*) gene, which is associated with retinal degeneration. Therefore, in ocular research, it would not be appropriate to choose B6N as a background or *in vivo* model, crossbreed it with B6J, or compare it with B6J. Similarly, these substrain differences must be considered when selecting mouse substrains in metabolic studies.

Many phenotypic differences [11–24] between B6J and B6N, especially in metabolic traits such as glucose tolerance, insulin secretion, and body weight, have been attributed to loss of function mutations in the nicotinamide nucleotide transhydrogenase *(Nnt*) gene, including deletion of five exons and a missense mutation within the *Nnt* locus, which is harbored only in B6J mice [11, 25, 26]. However, conflicting data that do not correlate with *Nnt* mutations exist for differences in body weight between the two substrains, with reports of B6J mice weighing more than B6N mice [12–14, 27, 28], B6J mice weighing less than B6N mice [15, 29–32], and no difference in body weight between the two substrains [16, 17, 33]. In addition, conflicting data also exist regarding whether glucose tolerance and insulin secretion are impaired in B6J or B6N mice [12–19, 29, 31, 33]. These contradictory reports suggest that *Nnt* is not the only genetic variant that needs to be considered, and other unknown variants that affect metabolic traits in these substrains need to be ascertained.

In this study, we aimed to investigate the genetic causes of the phenotypic differences between B6J and B6N substrains other than *Nnt* by comparing mice with spontaneous lack of *Nnt* and *Nnt*-wild type mice. Metabolic phenotypic differences between B6J and B6N substrains were characterized by examining blood components involved in metabolism, energy balance, and body and tissue weight. Determining consistent genetic differences across multiple tissues involved in metabolic traits such as subcutaneous and visceral white adipose tissues, brown adipose tissue, muscle, liver, and brain may help explain phenotypic differences in metabolism between the two substrains. Considering the extremely wide use of both substrains and their critical importance in generating transgenic and knock-out models, these findings guide future research across several interrelated fields.

## Materials and methods

### Animals and experimental design

All experimental procedures were approved and performed in accordance with the Institutional Animal Care and Use Committee of the RIKEN Yokohama Campus. Seven-week- old male C57BL/6J and C57BL/6NCrl mice were purchased from the Oriental Yeast Company, Ltd. (Shiga, Japan). Genotyping for nicotinamide nucleotide transhydrogenase (*Nnt*) in both substrains confirmed that only B6J lacks NNT (S1 Fig). Mice were acclimated for 1 week and maintained on an alternating 12 h light/dark cycle at a temperature of 23 °C, with free access to food and water. After the acclimatization period, the mice were randomly divided into two experimental groups per strain (n = 5 per group): normal diet (ND) (CLEA Rodent Diet CE-2; CLEA Japan Inc., Shizuoka, Japan) or a high-fat diet (HF) (High-Fat Diet 32; CLEA Japan Inc.) for 30 weeks. The basic diet composition is shown in S1 Table. At the age of 20–30 weeks old, a metabolic assessment was performed. At the age of 38 weeks old, mice were euthanized under isoflurane anesthesia, and blood and organs were collected. Inguinal white adipose tissue (iWAT), epididymal white adipose tissue (eWAT), brown adipose tissue (BAT), skeletal soleus muscle (muscle), liver, hypothalamus (Hyt), and hippocampus (Hic) were rapidly removed, weighed, and submerged in RNAlater solution (Thermo Fisher Scientific, Waltham, MA, USA) at 4 °C for 20 h, and stored at –20 °C. The timeline of the experiment is demonstrated in S2 Fig.

### Plasma parameters

Blood glucose levels were determined using a compact glucose analyzer (Glutest Sensor; Sanwa Kagaku, Nagoya, Japan). Plasma insulin (Morinaga Institute of Biological Science, Kanagawa, Japan), leptin (R&D Systems, Minneapolis, MN, USA), and adiponectin (Otsuka Pharmaceutical Co., Ltd., Tokyo, Japan) levels were measured using an ELISA kit. Plasma triglyceride (TG), total cholesterol (T-Cho), high-density lipoprotein cholesterol (HDL), non-esterified fatty acids (FFA), alanine aminotransferase (ALT), aspartate aminotransferase (AST), and lactate dehydrogenase (LDH) levels were measured using reagents from Wako Pure Chemical Industries, Ltd. (Osaka, Japan). All assays were performed according to the manufacturer’s instructions.

### Metabolic assessments

Oxygen consumption (VO_2_) and carbon dioxide exhalation (VCO_2_) were measured using an open-circuit metabolic gas analysis system connected directly to a mass spectrometer (ARCO-2000; Arco Systems Inc., Chiba, Japan). The mice were housed in individual acrylic chambers with free access to food and water. After five days of acclimation, in which food intake and VCO_2_/VO_2_ values stabilized, data were recorded in individual mice for 1 min at 15 min intervals over a 7-day period, and mean values for every 12-h were used for the analysis. The measurement protocol including the airflow (0.3 L/min) and data acquisition interval was determined to measure 10 mouse chambers simultaneously along with the calibration gas line. Total energy expenditure was calculated based on Lusk’s equation [34]. Carbohydrate and fat consumption were calculated based on Frayn’s equation [35]. Energy expenditure = 3.816 × VO_2_ + 1.231 × VCO_2_ [kcal]. Total carbohydrate consumption = 4.585 × VCO_2_ − 3.226 × VO_2_ [mg.min^−1^]. Fat consumption = 1.695 × VO_2_ − 1.701 × VCO_2_ [mg.min^−1^]. Data were normalized to body weight. Locomotor activity was estimated based on the number of infrared beams broken in both the x- and y-directions using an activity monitoring system combined with a food intake recording system (ACTIMO-100M/MFD-100M; Shin Factory, Fukuoka, Japan).

### RNA sequencing

#### Tissue preparation and RNA isolation

Minced tissues were homogenized with Sepasol RNAI solution (Nacalai Tesque, Kyoto, Japan) using a TissueLyser LT instrument (Qiagen, Hilden, Germany) set at 50 strokes/s for 5 min. The homogenate from adipose tissues was centrifuged at 3000 × *g* for 10 min, and the bottom layer was transferred into a new tube to separate the fat from the upper layer. Chloroform was then added to the sample, and the vortexed sample was centrifuged at 14000 × *g* for 10 min to separate the RNA phase. The RNA phase was then transferred to a new tube and subjected to total RNA purification using QIAcube and the RNeasy kit (Qiagen). Quality analysis of RNA samples was performed using TapeStation (Agilent Technologies, Santa Clara, CA, USA) and RNA ScreenTape (Agilent Technologies).

#### Library construction and sequencing

Libraries were generated with the NEBNext Ultra RNA Library Prep Kit for Illumina (New England Biolabs, Ipswich, MA, USA). mRNA was enriched from total RNA (250 ng) using magnetic poly-T beads. First- and second-strand cDNAs were synthesized using random hexamer primers, M-MuLV reverse transcriptase, DNA polymerase I, and RNase H, followed by the conversion of overhangs to blunt ends. DNA fragments were ligated with NEBNext adaptors and size-fractionated with the AMPure XP system (Beckman Coulter, Inc., CA, USA) before treatment with the USER enzyme (New England Biolabs) and polymerase chain reaction (PCR) amplification with universal and index primers using Phusion high-fidelity DNA polymerase. PCR products were purified using the AMPure XP system, and the quality of the library was assessed using the TapeStation system (Agilent Technologies). Pooled libraries were sequenced on an Illumina HiSeq 2500 platform to obtain 50 bp single-end reads.

#### Read mapping and quantification of gene expression level

Reads were mapped to genes in the reference mouse genome (UCSC mm9) and assembled into transcripts, whose abundance was estimated as the expected number of fragments per kilobase per million base pairs sequenced (FPKM) using Cufflinks (v 1.3.0). Bowtie (v 0.12.7) was used to build an index of the reference genome, and TopHat (v 1.4.0) was used to align the reads.

#### Differential gene expression analysis

Data were analyzed using Strand NGS (v. 2.7, Strand Life Sciences, Bengaluru, India). DESeq was used to compare pairs of sample groups that included five biological replicates for each group. All genes were tested to obtain the corresponding *p*-values, followed by multiple testing corrections using the Benjamini and Hochberg method to acquire the corrected *p*-value (*q*). Comparing the significance of differences in gene expression levels between groups was analyzed using Tukey’s post hoc test on normalized FPKM.

### Statistical analysis

Statistical analyses were performed using GraphPad Prism 8 software. Quantitative two-group data were analyzed using an unpaired two-tailed *t*-test. A comparison of data with two and three factors was performed using an analysis of variance ANOVA.

## Results

### B6J mice have lower body weight than B6N mice

**Fig 1:**
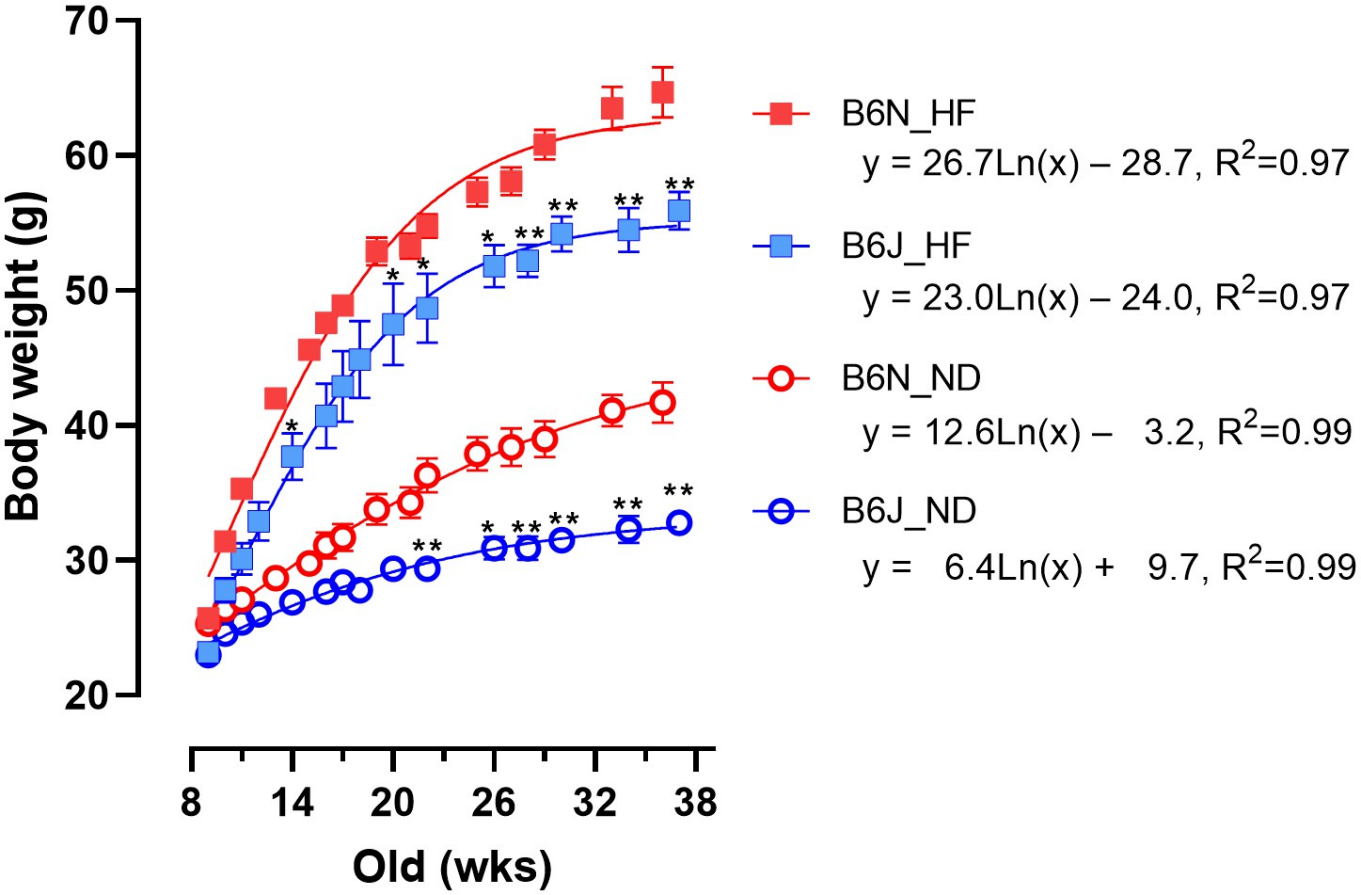
Difference in body weight between B6J and B6N mice on ND or HF. The body weight of male B6J and B6N mice on ND or HF was monitored up to 38 weeks of age (n = 5 per group). Data are presented as mean ± SEM. Asterisks (*) denote significant differences (* *p*<0.05, ** *p*<0.01) between B6J and B6N mice at each time point in the same food group. The lines and the equation were based on regression analysis (log-ratio transformed). ND, normal diet; HF, high-fat diet; open circles (blue), ND-fed B6J; open circles (red), ND-fed B6N; filled squares (blue), HF-fed B6J; filled squares (red), HF-fed B6N.

There were considerable differences in body weight between the B6J and B6N groups, regardless of whether they were fed ND or HF (Fig 1). Regression analysis showed that B6J mice gained less weight than B6N mice 2-fold in the ND group and 1.2-fold in the HF group. Previously, there have been conflicting findings as to whether B6J or B6N mice are more likely to gain weight [12–17, 27–33]. The present results corroborate with prior studies showing that B6N mice were more likely to gain weight [15, 29–32].

### B6J mice exhibit lower adiposity than B6N mice

**Fig 2:**
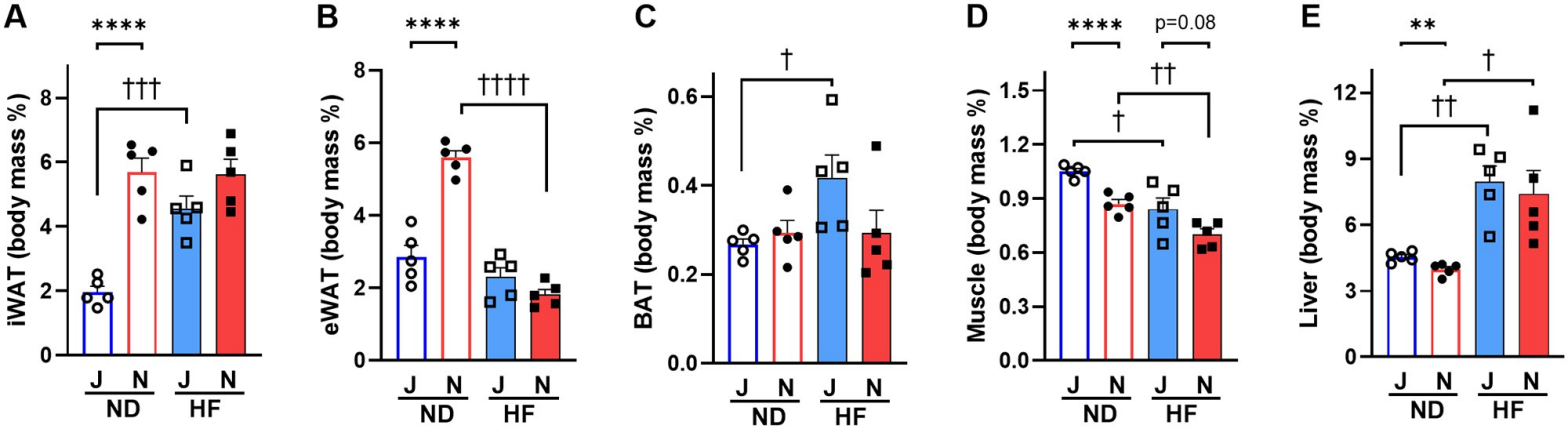
Tissue weights in B6J and B6N mice on ND or HF. Relative tissue weights were calculated by organ weight/body weight (body mass percentage). (A) iWAT, inguinal white adipose tissue; (B) eWAT, epididymal white adipose tissue; (C) BAT, brown adipose tissue; (D) Muscle, skeletal muscle; and (E) Liver. Values are means + SEM (n = 5) for B6J group (blue color) and B6N group (red color). Two-way ANOVA indicated a significant interaction between substrain and diet for iWAT (*p*<0.01; F = 11.96; Df = 16) and eWAT (*p* <0.0001; F = 47.48; Df = 16). There was a significant effect of strain on muscle (*p*<0.001; F = 18.46; Df = 16) independent of diet, and significant effect of diet on muscle (*p*<0.001; F = 25.63; Df = 16) and on liver (*p*<0.0001; F = 27.80; Df = 16) independent of strain. Individual means were compared within groups using unpaired Student’s *t*-test. Asterisks (*) and daggers (†) indicate significant differences between B6J and B6N substrain groups, and ND and HF diet groups, respectively (** *p*<0.01 and **** *p*<0.0001, † *p* <0.05, †† *p* <0.01, ††† *p* <0.001, and †††† *p*<0.0001). J, B6J substrain; N, B6N substrain; ND, normal diet; HF, high-fat diet; open circles, ND-fed B6J; filled circles, ND-fed B6N; open squares, HF-fed B6J; filled squares, HF-fed B6N.

Substrain differences in tissue weight were found in white adipose tissues, muscle, and liver of the ND-fed group. The weights of the white adipose tissues (iWAT and eWAT) of ND-fed B6J mice were significantly (*p*<0.0001 for both iWAT and eWAT) less than those of B6N mice, when measured in absolute weight (S3A and S3B Fig) and when normalized to body weight (Fig 2A and 2B). Liver and muscle weights were slightly less in absolute weight in ND-fed B6J mice (S3D and S3E Fig); however, liver and muscle weights normalized to body weight were slightly higher in B6J mice (Fig 2D and 2E) compared with those in B6N mice. In the group of mice fed HF, the iWAT weight of B6J mice tended to be lower than that of B6N mice (Fig 2A and S3A Fig), whereas eWAT was similar (Fig 2B and S3B Fig). The relative weight of BAT of HF-fed B6J mice tended to be higher than that of B6N mice (Fig 2C). Similarly, B6J mice fed HF tended to have higher relative weight of liver and muscle than B6N mice (Fig 2E and 2D). Looking at the effect of the HF diet on tissue weight, HF caused a significant (*p* = 0.011 for B6J and *p* = 0.003 for B6N) reduction in the muscle in both B6J and B6N mice (Fig 2D). In contrast, HF significantly (*p* = 0.002 for B6J and *p* = 0.013 for B6N) increased liver in both B6J and B6N mice (Fig 2E). HF also significantly (*p* = 0.0003 for iWAT and *p* = 0.025 for BAT) increased iWAT and BAT weights, but only in B6J mice, not in B6N mice (Fig 2A and 2C). Surprisingly, HF did not increase eWAT weight in both B6J and B6N mice; rather, eWAT weight in B6N mice was significantly (*p*<0.0001) less in the HF group than in the ND group (Fig 2B). Collectively, these results indicate that B6J mice have lower adiposity and more pronounced HF-induced increases in iWAT and BAT than B6N mice. Moreover, the increased fat retention in BAT in B6J mice suggests that metabolic strategies such as burning more energy or increasing the conversion of WAT to BAT may have resulted in less weight gain in B6J mice.

### Plasma leptin and adiponectin levels in B6J mice are lower than those in B6N mice

**Fig 3:**
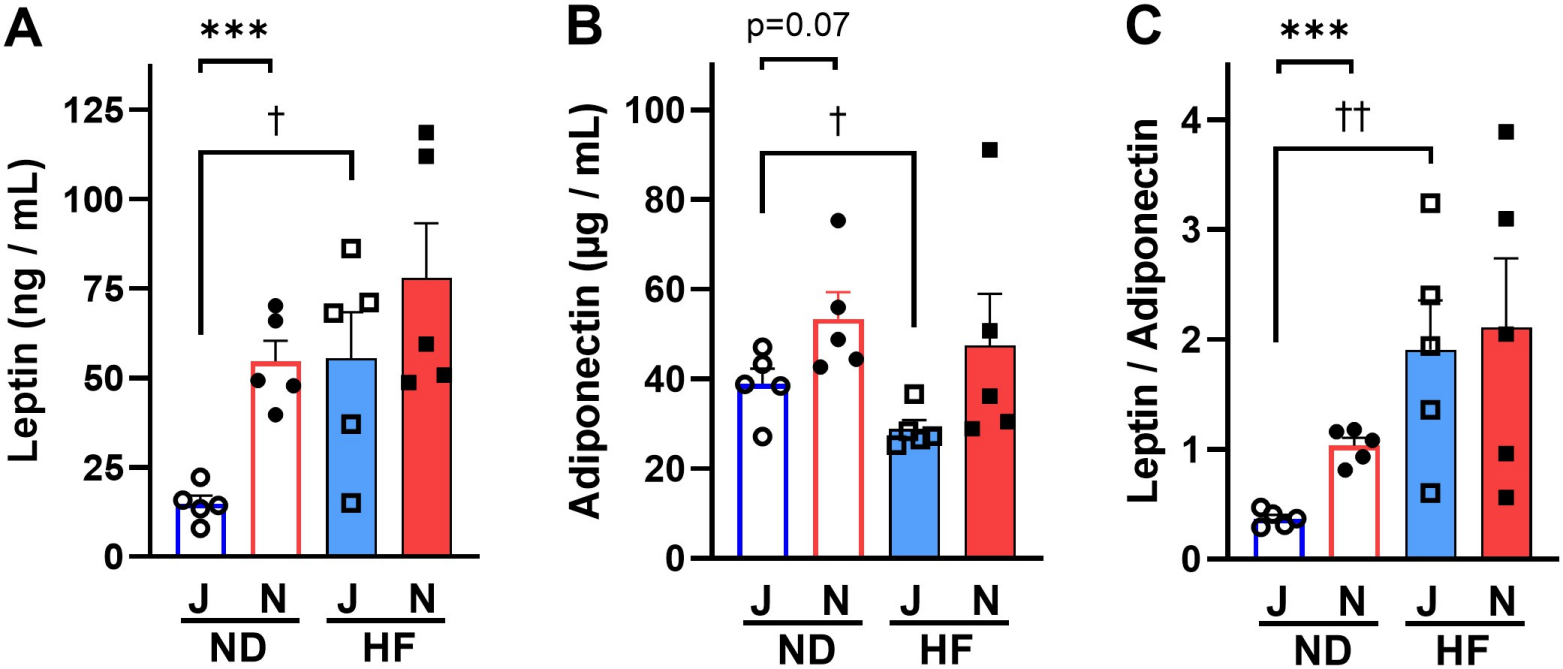
Differences in plasma leptin, adiponectin, and leptin to adiponectin ratio in B6J and B6N mice on ND or HF. (A) Leptin, (B) Adiponectin, (C) Leptin/Adiponectin. Bars are representative of means + SEM (n = 5) for B6J group (blue color) and B6N groups (red color). Two-way ANOVA indicated a significant effect of strain on leptin (*p* <0.01; F = 8.78; Df = 16) and adiponectin (*p*<0.05; F = 5.97; Df = 16) independent of diet, and a significant effect of diet on leptin (*p* <0.01; F = 9.29; Df = 16) and on leptin/adiponectin (*p* <0.01; F = 11.40; Df = 16) independent of strain. Individual means were compared within groups using unpaired Student’s *t*-test. Asterisks (*) and daggers (†) indicate significant differences between B6J and B6N substrain groups and ND and HF diet groups, respectively (*** *p* <0.001, ^†^ *p* <0.05, and ^††^ *p*<0.01). J, B6J substrain; N, B6N substrain; ND, normal diet, HF, high-fat diet; open circles, ND-fed B6J; filled circles, ND-fed B6N; open squares, HF-fed B6J; filled squares, HF-fed B6N.

To confirm the lower adiposity in B6J mice than in B6N mice (Fig 2A and S3A Fig), we measured two adipokines: leptin and adiponectin. In the ND-fed groups, the plasma leptin levels of B6J mice were significantly (*p* = 0.0002) lower than those of B6N mice (Fig 3A). The adiponectin levels were also lower in ND-fed B6J mice than in ND-fed B6N mice but in a slightly weaker trend (*p* = 0.07, Fig 3B). In the HF-fed groups, adipokine levels were also lower in B6J mice than in B6N mice; however, the difference was not statistically significant (Fig 3A and 3B). Considering that leptin increases with fat accumulation and adiponectin decreases, the leptin/adiponectin ratio correlates well with adiposity [36]. In addition, this ratio is correlated with insulin resistance, which may represent adipose tissue dysfunction [36]. Therefore, we calculated the ratio of leptin to adiponectin and found it to be approximately four-fold lower (*p* = 0.0002) in B6J mice than in B6N fed ND (Fig 3C), confirming that the B6J substrain has lower adiposity than B6N substrain.

### Plasma insulin levels of B6J mice are lower than those of B6N mice

**Fig 4:**
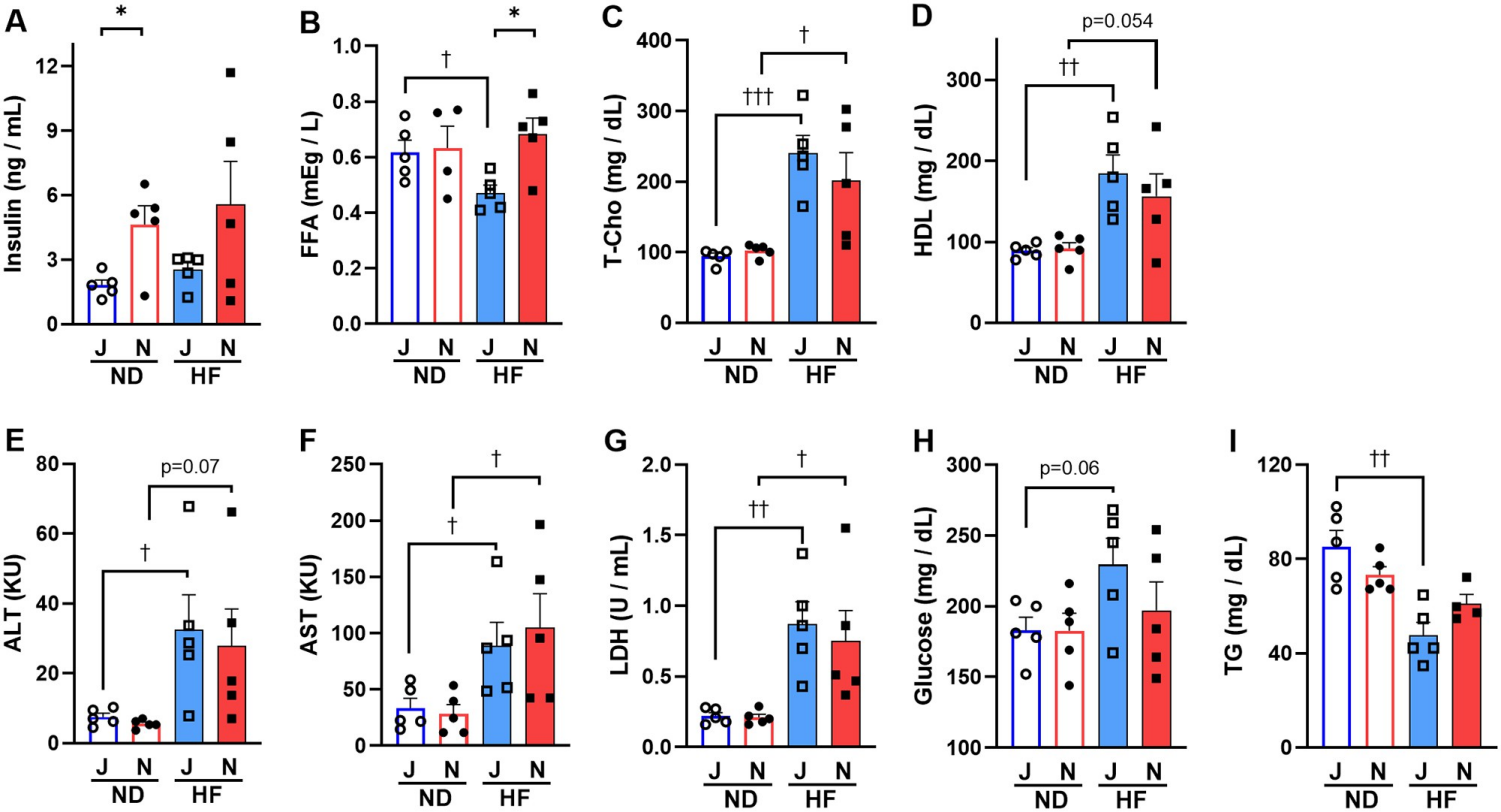
Differences in metabolic parameters in the plasma of B6J and B6N mice on ND or HF. (A) Insulin, (B) FFA, non-esterified fatty acids:, (C) T-Cho, total cholesterol;, (D) HDL, high-density lipoprotein cholesterol;, (E) ALT, alanine aminotransferase;, (F) AST, aspartate aminotransferase;, (G) LDH, lactate dehydrogenase;, (H) Glucose, and (I) TG, triglyceride. Bars are representative of means + SEM (n = 5), for B6J group (blue color) and B6N group (red color). Two-way ANOVA indicated a significant interaction between substrain and diet for TG (*p*<0.05; F = 5.89; Df = 15). There was a significant effect of strain on insulin (*p*<0.05; F = 6.87; Df = 16) and FFA (*p*<0.05; F = 4.67; Df = 15) independent of diet, and a significant effect of diet on T-Cho (*p*<0.0001; F = 27.50; Df = 16), HDL (*p*<0.001; F = 18.66; Df = 16), ALT (*p*<0.01; F = 11.83; Df = 16), AST (*p* <0.01; F = 10.74; Df = 16), LDH (*p* <0.001; F = 19.75; Df = 16), and TG (*p*<0.001; F = 22.85; Df = 15) independent of strain. Individual means were compared within groups using unpaired Student’s *t*-test. Asterisks (*) and daggers (†) indicate significant differences between B6J and B6N substrain groups and ND and HF diet groups, respectively (* *p*<0.05, ^†^ *p*<0.05, ^††^ *p*<0.01, and ^†††^ *p*<0.001). J, B6J substrain; N, B6N substrain; ND, normal diet, HF, high-fat diet; open circles, ND-fed B6J; filled circles, ND-fed B6N; open squares, HF-fed B6J; filled squares, HF-fed B6N.

We further compared commonly measured blood metabolic parameters, such as glycemic (glucose and insulin), lipidic (T-Cho, HDL, FFA, and TG), and liver enzymes (ALT, AST, and LDH). The only blood components that differed between B6J and B6N mice were insulin and FFA (Fig 4A and 4B). Insulin concentrations were significantly (*p* = 0.015) lower in B6J mice than in B6N mice under the ND fed condition (Fig 4A). FFA was significantly (*p* = 0.010) lower in HF-fed B6J mice than in HF-fed B6N mice (Fig 4B). T-Cho (Fig 4C), HDL (Fig 4D), ALT (Fig 4E), AST (Fig 4F), and LDH levels (Fig 4G) were not statistically different between B6J and B6N mice, in either diet group, whereas these levels were considerably higher in the HF groups than in the ND groups (Fig 4C–4G). Additionally, the blood glucose level in B6J mice and, not in B6N mice was higher in the HF group than in the ND group (Fig 4H). Similarly, TG concentration in B6J mice fed the HF diet were significantly (*p* = 0.003) lower than in mice fed the ND diet, with less reduction in B6N mice (Fig 4I).

### B6J mice consume more energy than B6N mice

**Fig 5:**
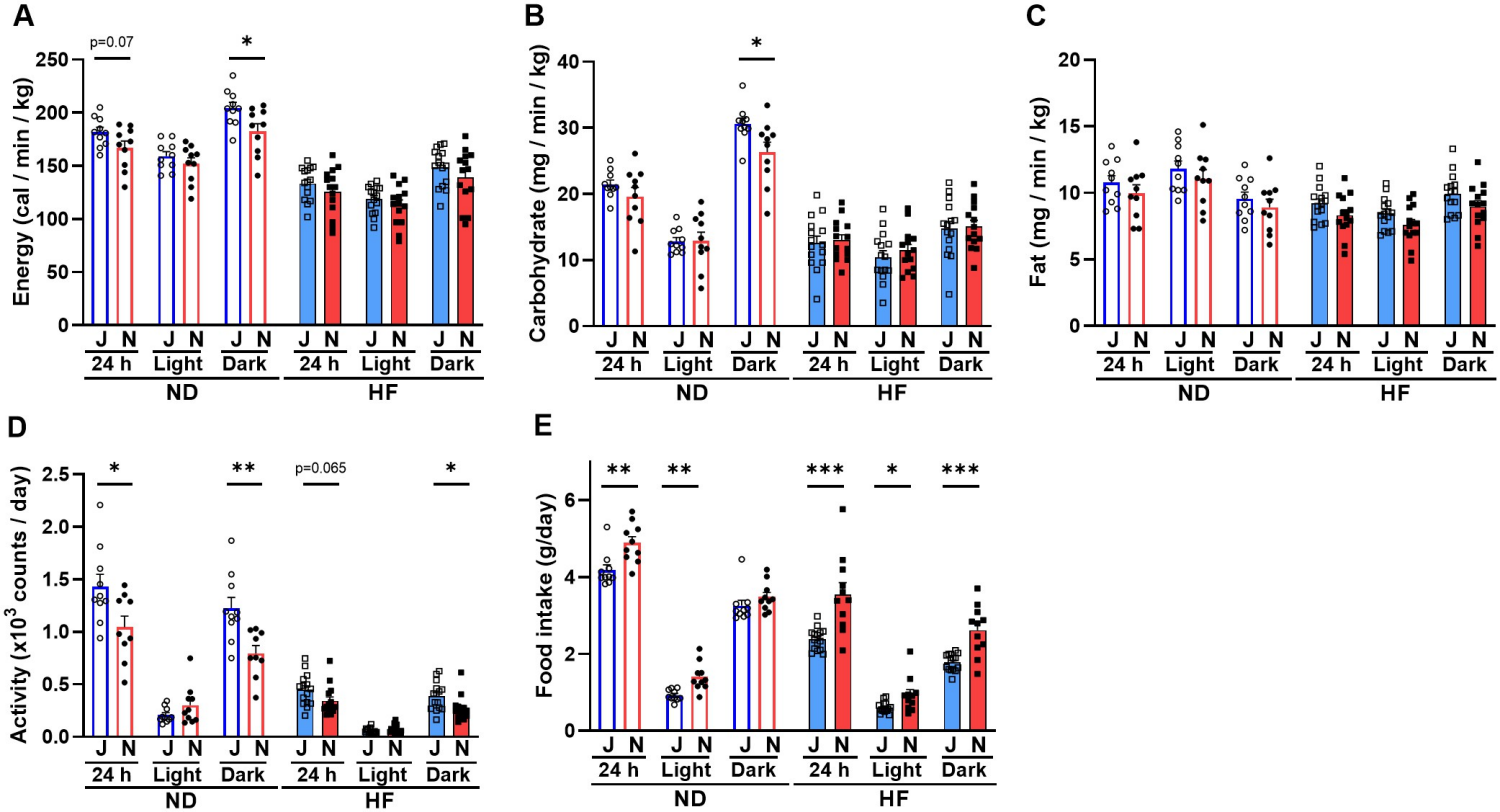
Differences in metabolic assessments between B6J and B6N mice. (A) Energy expenditure (B) Carbohydrate consumption (C) Fat consumption (D) Activity, and (E) Food intake. Values are means + SEM (n = 5, experimented in duplicates), and asterisks (*) indicate significant differences (* *p*<0.05, ** *p*<0.01, and *** *p*<0.001, unpaired Student’s *t*-test). Open circles, ND-fed B6J; filled circles, ND-fed B6N; open squares, HF-fed B6J; filled squares, HF-fed B6N.

To determine the difference in metabolic rates between B6J and B6N mice, we measured oxygen consumption and carbon dioxide production and calculated energy, carbohydrate, and fat consumptions (normalized to body weight). Values are presented per diet and per light/dark phase (“Dark” for the active phase and “Light” for the inactive phase, “24 h” throughout both phases). Although the overall differences between B6J and B6N mice were not large, some marked differences were observed such as energy expenditure, with B6J mice tending to expend more energy than B6N mice during the active phase (Fig 5A). Similarly, there was a notable difference in carbohydrate consumption when fed ND, with B6J mice consuming more carbohydrates than B6N mice during the active phase (Fig 5B). Fat consumption data also showed differences between B6J and B6N when fed HF, with B6J mice consuming more fat than B6N mice throughout the day (Fig 5C). In addition, behavioral factors that affect metabolic rates, such as physical activity and food intake, were assessed. B6J mice tended to be more active than B6N mice when fed both ND or HF (Fig 5D) and took considerably less food (Fig 5E).

### DEGs between B6J and B6N

**Fig 6:**
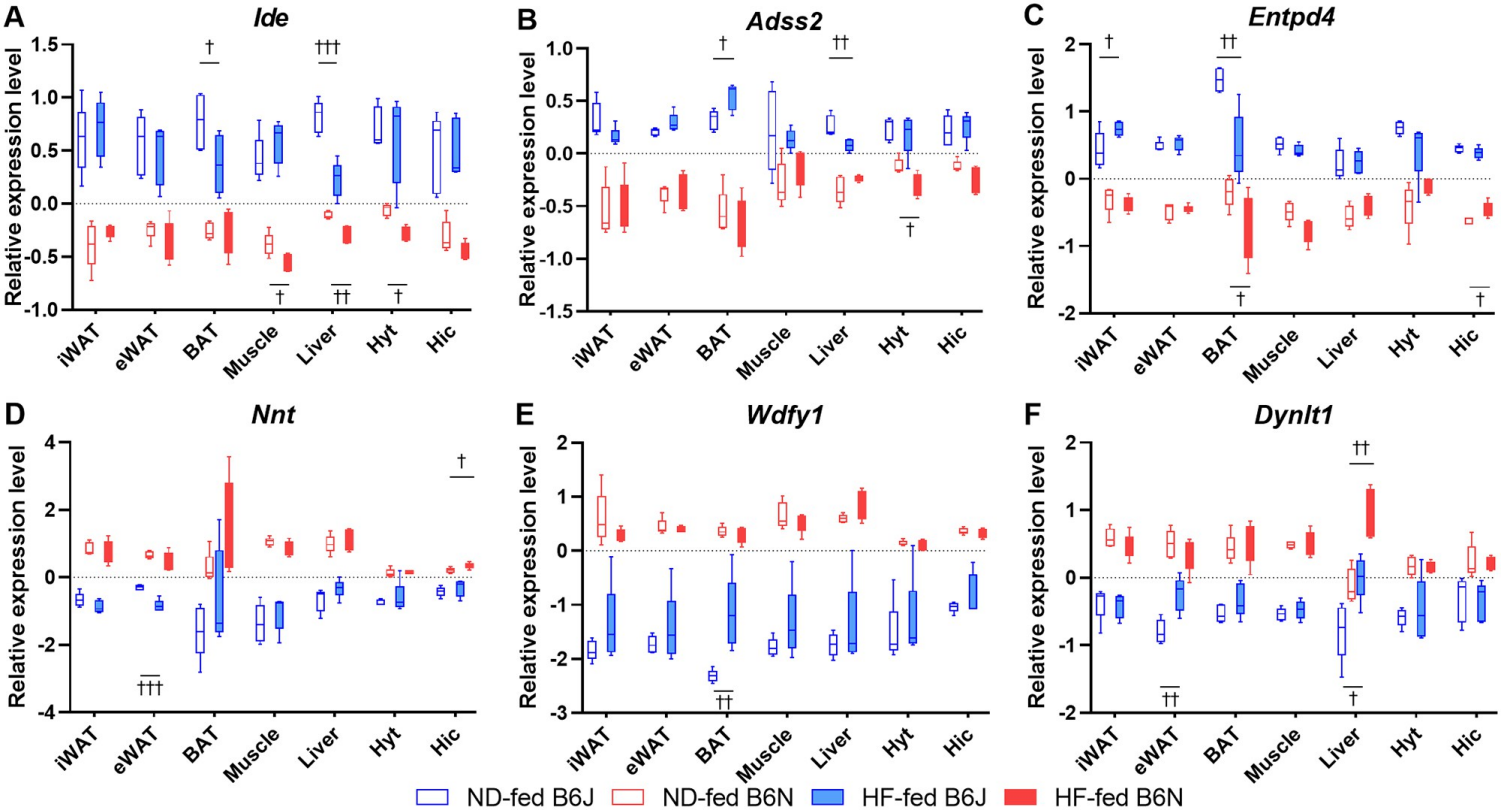
Expression levels of DEGs between B6J and B6N on ND or HF. The gene name is indicated at the top of each plot, and the y-axis represents the normalized signal values. (A) *Ide*, insulin degrading enzyme; (B) *Adss2*, adenylosuccinate synthase 2; (C) *Entpd4*, ectonucleotide triphosphate diphosphohydrolase 4; (D) *Nnt*, nicotinamide nucleotide transhydrogenase; (E) *Wdfy1*, WD repeat and FYVE domain containing 1; (F) *Dynlt1*, dynein light chain Tctex-type1. Boxes are representative of means ± SEM (n = 5) for the B6J (blue color) and B6N (red color) groups. Three-way ANOVA (Df = 110 for all analysis) indicated a significant interaction between substrain, diet, and tissue for *Adss2* (*p*<0.05; F = 2.59) and *Entpd4* (*p*<0.01; F = 3.24); a significant interaction between substrain and diet for *Wdfy1* (*p*<0.001; F = 13.96); a significant interaction between diet and tissue for *Ide* (*p*<0.05; F = 2.71), *Entpd4* (*p*<0.0001; F = 10.38), *Nnt* (*p*<0.001; F = 4.32), and *Dynlt1* (*p*<0.0001; F = 9.51) and a significant interaction between strain and tissue for *Adss2* (*p*<0.0001; F = 10.90), *Entpd4* (*p*<0.0001; F = 5.49), *Nnt* (*p*<0.0001; F = 6.75), *Wdfy1* (*p*<0.001; F = 4.34), and *Dynlt1* (*p*<0.05; F = 2.24). There was a significant effect of strain on *Ide*, *Adss2*, *Entpd4*, *Nnt*, *Wdfy1*, and *Dynlt1* (*p*<0.0001; F = 554, 453, 723, 289, 821, and 406, respectively) independent of diet and tissue, and a significant effect of diet on *Ide* (*p*<0.01; F = 10.40), *Entpd4* (*p*<0.05; F = 6.56), *Wdfy1* (*p*<0.01; F = 8.73), and *Dynlt1* (*p*<0.0001; F = 17.78) independent of strain and tissue. There was a significant effect of tissue on *Ide* (*p*<0.05; F = 2.39), *Adss2* (*p*<0.05; F = 2.53), *Entpd4* (*p*<0.0001; F = 8.65), *Nnt* (*p*<0.05; F = 2.31), *Wdfy1* (*p*<0.05; F = 2.54), and *Dynlt1* (*p*<0.05; F = 2.21) independent of strain and diet. Daggers (†) indicate significant differences between ND and HF groups (^†^ *p*<0.05, ^††^ *p*<0.01, and ^†††^ *p*<0.001, Tukey’s post hoc test). ND, normal diet; HF, high-fat diet; open blue boxes, ND-fed B6J; open red boxes, ND-fed B6N; filled blue boxes, HF-fed B6J; filled red boxes, HF-fed B6N.

Table 1 shows the number of DEGs (*p*<0.05) in the iWAT, eWAT, BAT, muscle, liver, hypothalamus, and hippocampus between B6J and B6N mice. Comparable numbers of DEGs were upregulated and downregulated in each tissue in the ND and HF groups, respectively. The gene names of the DEGs in each tissue are listed in S2 Table, along with their average fold change (FC), *p*-values, and corrected *p*-value (*q*). Among the DEGs that were highly expressed in B6J mice compared to B6N mice, three genes: insulin degrading enzyme (*Ide*), adenylosuccinate synthase 2 (*Adss2*), and ectonucleotide triphosphate diphosphohydrolase 4 (*Entpd4*) in the ND group and five genes: *Ide*, *Adss2*, *Entpd4*, B-TFIID TATA-box binding protein associated factor 1 (*Btaf1*), and transmembrane protein 267 (*TMEM267*) in the HF group overlapped in all seven tissues examined in the present study. Likewise, among the DEGs that showed lower expression in B6J mice compared with in B6N mice, four genes: *Nnt*, WD repeat and FYVE domain containing 1 (*Wdfy1*), dynein light chain Tctex-type 1 (*Dynlt1*), and RAB4A, member RAS oncogene family (*Rab4A*) in the ND group and three genes: *Nnt*, *Wdfy1*, and *Dynlt1* in the HF group were identified. Of these overlapping genes, *Wdfy1* showed high FC and was significantly (*q*<0.05, S3 Table) lower in ND-fed B6J mice by approximately 4- to 6-fold compared with in ND-fed B6N mice (Fig 6E). *Entpd4* also showed consistent FC in the HF group with approximately 2- to 3-fold higher expression in B6J mice than in B6N (Fig 6C).

**Table 1:** Number of differentially expressed genes (DEGs) in the iWAT, eWAT, BAT, muscle, liver, Hyt, and Hic between B6J and B6N mice fed a normal diet (ND) or a high-fat diet (HF).

		J > N	J < N			J > N	J < N
iWAT	ND	731	1349	Liver	ND	972	906
	HF	888	790		HF	768	629
	ND ∩ HF	29	31		ND ∩ HF	85	44
eWAT	ND	2450	2547	Hyt	ND	1037	1163
	HF	1190	753		HF	2109	1987
	ND ∩ HF	160	105		ND ∩ HF	186	132
Bat	ND	2001	2162	Hic	ND	1122	1099
	HF	2489	2110		HF	1177	929
	ND ∩ HF	259	336		ND ∩ HF	102	99
Muscle	ND	933	917	7 tissues	ND	3	4
	HF	1491	1448		HF	5	3
	ND ∩ HF	203	122		ND ∩ HF	3	3

DEGs were screened using the criteria of *p*<0.05 and fold change (FC) >1. J>N, the expression level in B6J was higher than in B6N; J<N, the expression level in B6J was lower than in B6N; ND∩HF, the number of overlapping DEGs among ND-fed and HF-fed mice. iWAT, inguinal white adipose tissue; eWAT, epididymal white adipose tissue; BAT, brown adipose tissue; Muscle, skeletal muscle; Hyt, hypothalamus; Hic, hippocampus; ND, normal diet; HF, high-fat diet.

The effect of a high-fat diet on the expression of these overlapping genes was not observed in most tissues. However, decreased *Ide* expression and increased *Dynlt1* expression in the liver (Fig 6A and 6F) and decreased *Entpd4* expression in BAT (Fig 6C) were observed under HF conditions compared to those observed under ND conditions.

## Discussion

Phenotypic differences among B6 substrains have been reported in many studies in a variety of fields, including metabolism [7, 12, 14–19, 31, 33], alcohol preference [37–40], stress response [27, 32, 41–46], cardiovascular [20, 21, 47], oxidative stress [22, 23], vision [8–10], bone [28], liver [29, 30, 48], kidney [24, 49, 50], microbiota [15], and seizures [51]. However, the genes responsible for these phenotypic differences have not been identified. Often, NNT deficiency due to a spontaneous missense mutation of *Nnt* in B6J mice is considered to be responsible for these phenotypic differences and abnormal traits in B6J mice [11–24], including metabolic dysfunctions such as impaired glucose tolerance, diminished insulin secretion, and overweightness. This speculation is based on the fact that the enzymatic function of NNT is to pump protons across the inner mitochondrial membrane [26]. However, the difference in the phenotype between B6J and B6N substrain is not always correlated to the absence or presence of NNT. For example, *Nnt*-deficient B6J mice (S1 Fig) in the present study were considerably underweight compared with B6N mice (Fig 1), as reported by previous studies [15, 29–32]. However, there are conflicting data on body weight differences: overweight B6J mice [12–14, 27, 28] or unchanged weight [16, 17, 33]. Similarly, there are also conflicting data on the differences in blood insulin and glucose levels between B6J and B6N mice with several studies reporting that B6J mice have lower insulin levels [12–14, 17–19, 29, 31], higher glucose levels [12, 15, 18, 19, 31], and lower glucose levels [14] compared to B6N mice, whereas some studies did not report any differences in glucose [16, 17, 29, 31, 33] or insulin [16, 31] levels. In our study, we observed higher glucose levels but lower insulin levels in *Nnt*-deficient B6J mice compared with *Nnt*-wild type B6N mice (Fig 4). Moreover, literature on DEGs between B6N, B6J, and B6J expressing the full-length *Nnt* gene (B6JBAC) in the adrenal, testes, and heart has shown that there were no common DEGs across tissues when comparing the B6J and B6JBAC mice, despite the presence of far more DEGs in both compared with in B6N mice, suggesting a very modest effect of NNT status on transcriptome regulation and phenotype differences [52]. Given the discrepancy between the B6J and B6N phenotypes and NNT levels, we speculate that genetic mutations other than *Nnt* mutation alleles are present in either B6J or B6N substrains.

We investigated DEGs between B6J and B6N substrains in seven tissues involved in metabolic function: iWAT, eWAT, BAT, muscle, liver, hypothalamus, and hippocampus tissues, assuming that, besides *Nnt*, there are common genes across tissues regulating metabolic phenotypes that may explain the substrain differences. As a result, six DEGs, including *Nnt*, overlapped in all seven tissues regardless of dietary conditions (Fig 6). Three DEGs: *Ide*, *Adss2*, and *Entpd4* were significantly (*p*<0.05) more expressed in B6J mice than in B6N mice, whereas three DEGs: *Nnt*, *Wdfy1*, and *Dynlt1* were significantly (*p*<0.05) less expressed in B6J mice compared with B6N mice.

Previously, *Wdfy1* and *Entpd4* have been reported to be differentially expressed in the brain and pancreas between B6J and B6N [39, 53]; thus, our findings are consistent with those of this study. High *Wdfy1* expression in the brain of B6N is speculated to be related to reduced alcohol intake in B6N mice [39], whereas low *Wdfy1* expression in the pancreas of B6J mice is associated with the progression of chronic pancreatitis in B6J mice [53]. Although these previous studies did not focus on *Entpd4* [39, 53], its higher expression in B6J mice compared with that in B6N mice is consistent with the finding of the present study.

Watkins-Chow and Pavan have reported that the presence of increased copy number variation (CNV) at the *Ide* locus in B6J mice results in increased *Ide* expression [54]. The CNV may contribute to the divergence in *Ide* expression between B6J and B6N observed in this study. The lists of B6N substrains of protein-inactivating sequence variations (sequence variations causing premature stop codons, loss of stop codons and SNPs, and short in-frame insertions and deletions) that referenced the B6J mouse genome [55] did not include the *Ide*, *Adss2*, *Entpd4*, *Wdfy1*, and *Dynlt1* genes identified in this study. Therefore, CNVs in B6J and B6N mice should be investigated in addition to indels and SNPs [56]. Additionally, IDE is an enzyme that degrades insulin. Considering the lower blood insulin concentration and slightly higher glucose concentration in B6J mice compared with those in B6N mice (Fig 4A and 4H), the higher *Ide* expression level in B6J mice is consistent with the lower blood insulin concentration (Fig 6A). These results suggest that *Ide* may be responsible for the phenotypic differences in insulin secretion between B6J and B6N mice. *Ide* also plays a role in type 2 diabetes and Alzheimer’s disease [57]; thus, caution should be exercised when using B6 substrains in studies targeting these diseases.

A previous study has reported that mice lacking nucleobindin 2 (*NUCB2*), a precursor of nesfatin involved in appetite regulation, exhibit insulin resistance and high *Wdfy1* expression in their visceral macrophages [58], suggesting that *Wdfy1* may be also involved in insulin signaling. However, since the genetic background of the floxed mice used in the *NUCB2* paper [58] was B6J, and that of the crossbred recombinase mice was “C57BL/6J; C57BL/6N” [59], we cannot rule out the possibility that high *Wdfy1* expression was simply due to comparing B6N, which has high *Wdfy1* expression, with B6J, which has low expression. Similarly, the use of mice with a mixed background of B6J and B6N in immunological studies leads to confounding result interpretations. For example, the effect of granzyme A (GZMA) on viral arthritis remains inconclusive because the phenotypes observed in experiments using *GZMA* knockout mice vary from paper to paper [60]. However, the genetic background of *GZMA* knockout mice was found to be a mix of B6J and B6N, indicating that viral arthritis ameliorated in *GZMA* knockout mice was not the consequence of loss of GZMA expression, but rather of the genetic background of B6N, including *Nnt* [60].

To the best of our knowledge, no literature on *Adss2* and *Dynlt1* as DEGs between B6J and B6N mice currently exists. Nevertheless, we have identified DEGs between B6J and B6N (S2 Table), which may help plan mouse experiments. One limitation of this paper is that it lacks the potential to generalize the substrain differences due to the small sample size and the fact that only male mice were selected. Furthermore, gene expression analysis was conducted using an old version of the mouse genome mm9. However, in the DEG analysis between the two substrains, the well-known *Nnt* was also retrieved as a persistent gene across tissues, and *q* values for the six genes found here were below 0.1 in most of the conditions: seven tissues and two diets (S3 Table). This suggests that the genotypic data in this study are likely to be applicable and reliable. Our findings may be useful for revisiting past studies that have used B6J, B6N, or B6 substrains to determine whether their results answer the original research objectives or are merely a measurement of the differences between B6J and B6N.

## Conclusion

*Nnt* mutations in B6J mice have been implicated as a cause of obesity and various metabolic abnormalities and are described as the causative genetic explanation for the phenotypic differences between B6J and B6N mice [11–24]. However, there is a discrepancy in metabolic traits between B6J and B6N that cannot be explained by *Nnt* alone, raising the possibility that mutant alleles other than *Nnt* exist. We have identified persistent differentially expressed genes between B6J and B6N mice across all seven regions and two diets. Because these genes are associated with several diseases, including type 2 diabetes and Alzheimer’s, perhaps B6J and B6N substrains are unsuitable models for these diseases. Considering the extremely wide use of both substrains and their critical importance in generating transgenic and knock-out models, our findings help guide future research across several interrelated fields.

## Supporting information

S1 Table Basic diet composition of the normal diet (ND) and high-fat diet (HF). (TIF)

S2 Table Differentially expressed genes between B6J and B6N mice fed a normal diet or a high-fat diet. *P-*values were calculated using the moderated *t*-test, followed by multiple testing corrections using the Benjamini-Hochberg method to acquire the corrected *p*-value (*q*). Fold change (FC) values were calculated as log2 of B6J relative to B6N.
(XLSX)

S3 Table Differentially expressed genes between B6J and B6N mice overlapped across the tissues. *P-*values were calculated using the moderated *t*-test, followed by multiple testing corrections using the Benjamini-Hochberg method to acquire the corrected *p*-value (*q*). Fold change (FC) values were B6J relative to B6N. ND, normal diet; HF, high-fat diet; iWAT, inguinal white adipose tissue; eWAT, epididymal white adipose tissue; Bat, brown adipose tissue; Muscle, skeletal muscle; Hyt, hypothalamus; Hic, hippocampus.
(TIF)

S1 Fig Genotyping of the *Nnt* gene. Polymerase chain reaction analysis of *Nnt* alleles. DNA was obtained from the tail of B6J and B6N mice. The amplification products were 579 bp and 743 bp for the wild-type and mutant alleles, respectively.
(TIF)

S2 Fig Timeline showing the sequence of events the mice underwent. Four groups of animals were used. Colored boxes represent mouse substrain and diet groups; open blue box, normal diet (ND)-fed C57BL/6J (B6J); open red box, ND-fed C57BL/6NCrl (B6N); filled blue box, high-fat diet (HF)-fed B6J; filled red box, HF-fed B6N. Body weight was measured at the ages indicated by arrows. Mice were subjected to an indirect calorimetry system at the time points indicated as “metabolic assessment”. All animals were sacrificed and samples were taken at 38 weeks of age.
(TIF)

S3 Fig Absolute tissue weights in B6J and B6N mice on ND or HF. (A) iWAT, inguinal white adipose tissue; (B) eWAT, epididymal white adipose tissue; (C) BAT, brown adipose tissue; (D) Muscle, skeletal muscle; (E) Liver. Bars are representative of means + SEM (n = 5) for the B6J (blue color) and B6N (red color) groups. Two-way ANOVA indicated a significant interaction between substrain and diet for eWAT (*p*<0.0001; F = 38.56; Df = 16). There was a significant effect of strain on iWAT (*p*<0.0001; F = 27.49; Df = 16) independent of diet, and significant effect of diet on iWAT (*p*<0.01; F = 15.61; Df = 16), BAT (*p* <0.01; F = 9.50; Df = 16) and liver (*p*<0.001; F = 18.48; Df = 16) independent of strain. Individual means were compared within groups by unpaired Student’s *t*-test. Asterisks (*) and daggers (†) indicate significant differences between B6J and B6N substrain groups, and ND and HF diet groups, respectively (** *p*<0.01 and **** *p*<0.0001, ^†^ *p*<0.05, ^††^ *p*<0.01, and ^††††^ *p*<0.0001). J, B6J substrain; N, B6N substrain; ND, normal diet; HF, high-fat diet; open circles, ND-fed B6J; filled circles, ND-fed B6N; open squares, HF-fed B6J; filled squares, HF-fed B6N.
(TIF)

## References

[1] Mouse Genome Sequencing Consortium, Waterston RH, Lindblad-Toh K, Birney E, Rogers J, Abril JF, et al. Initial sequencing and comparative analysis of the mouse genome. Nature. 2002;420(6915):520–562. doi: 10.1038/nature01262 12466850

[2] Birling MC, Yoshiki A, Adams DJ, Ayabe S, Beaudet AL, Bottomley J, et al. A resource of targeted mutant mouse lines for 5,061 genes. Nat Genet. 2021;53(4):416–419. doi: 10.1038/s41588-021-00825-y 33833456 PMC8397259

[3] Mekada K, Yoshiki A. Substrains matter in phenotyping of C57BL/6 mice. Exp Anim. 2021;70(2):145–160. doi: 10.1538/expanim.20-0158 33441510 PMC8150240

[4] Åhlgren J, Voikar V. Experiments done in Black-6 mice: what does it mean? Lab Anim (NY). 2019;48(6):171–180. doi: 10.1038/s41684-019-0288-8 31011223

[5] Dobrowolski P, Fischer M, Naumann R. Novel insights into the genetic background of genetically modified mice. Transgenic Res. 2018;27(3):265–275. doi: 10.1007/s11248-018-0073-2 29663254

[6] Fontaine DA, Davis DB. Attention to background strain is essential for metabolic research: C57BL/6 and the International Knockout Mouse Consortium. Diabetes. 2016;65(1):25–33. doi: 10.2337/db15-0982 26696638 PMC4686949

[7] Simon MM, Greenaway S, White JK, Fuchs H, Gailus-Durner V, Wells S, et al. A comparative phenotypic and genomic analysis of C57BL/6J and C57BL/6N mouse strains. Genome Biol. 2013;14(7):R82. doi: 10.1186/gb-2013-14-7-r82 23902802 PMC4053787

[8] Lajko M, Cardona HJ, Taylor JM, Farrow KN, Fawzi AA. Photoreceptor oxidative stress in hyperoxia-induced proliferative retinopathy accelerates rd8 degeneration. PLoS One. 2017;12(7):e0180384. doi: 10.1371/journal.pone.0180384 28671996 PMC5495396

[9] Mattapallil MJ, Wawrousek EF, Chan CC, Zhao H, Roychoudhury J, Ferguson TA, et al. The Rd8 mutation of the Crb1 gene is present in vendor lines of C57BL/6N mice and embryonic stem cells, and confounds ocular induced mutant phenotypes. Invest Ophthalmol Vis Sci. 2012;53(6):2921–2927. doi: 10.1167/iovs.12-9662 22447858 PMC3376073

[10] Pak JS, Lee EJ, Craft CM. The retinal phenotype of Grk1-/- is compromised by a Crb1 rd8 mutation. Mol Vis. 2015;21:1281–1294. 26664249 PMC4663191

[11] Freeman HC, Hugill A, Dear NT, Ashcroft FM, Cox RD. Deletion of nicotinamide nucleotide transhydrogenase: a new quantitive trait locus accounting for glucose intolerance in C57BL/6J mice. Diabetes. 2006;55(7):2153–2156. doi: 10.2337/db06-0358 16804088

[12] Nicholson A, Reifsnyder PC, Malcolm RD, Lucas CA, MacGregor GR, Zhang W, et al. Diet-induced obesity in two C57BL/6 substrains with intact or mutant nicotinamide nucleotide transhydrogenase (*Nnt*) gene. Obesity (Silver Spring). 2010;18(10):1902–1905. doi: 10.1038/oby.2009.477 20057372 PMC2888716

[13] Anderson AA, Helmering J, Juan T, Li CM, McCormick J, Graham M, et al. Pancreatic islet expression profiling in diabetes-prone C57BLKS/J mice reveals transcriptional differences contributed by DBA loci, including *Plagl1* and *Nnt*. Pathogenetics. 2009;2(1):1. doi: 10.1186/1755-8417-2-1 19161594 PMC2642818

[14] Hull RL, Willard JR, Struck MD, Barrow BM, Brar GS, Andrikopoulos S, et al. High fat feeding unmasks variable insulin responses in male C57BL/6 mouse substrains. J Endocrinol. 2017;233(1):53–64. doi: 10.1530/JOE-16-0377 28138002 PMC5358546

[15] Smoczek M, Vital M, Wedekind D, Basic M, Zschemisch NH, Pieper DH, et al. A combination of genetics and microbiota influences the severity of the obesity phenotype in diet-induced obesity. Sci Rep. 2020;10(1):6118. doi: 10.1038/s41598-020-63340-w 32273571 PMC7145845

[16] Wong N, Blair AR, Morahan G, Andrikopoulos S. The deletion variant of nicotinamide nucleotide transhydrogenase (*Nnt*) does not affect insulin secretion or glucose tolerance. Endocrinology. 2010;151(1):96–102. doi: 10.1210/en.2009-0887 19906813

[17] Attané C, Peyot ML, Lussier R, Zhang D, Joly E, Madiraju SR, et al. Differential insulin secretion of high-fat diet-fed C57BL/6NN and C57BL/6NJ mice: Implications of mixed genetic background in metabolic studies. PLoS One. 2016;11(7):e0159165. doi: 10.1371/journal.pone.0159165 27403868 PMC4942110

[18] Fergusson G, Ethier M, Guévremont M, Chrétien C, Attané C, Joly E, et al. Defective insulin secretory response to intravenous glucose in C57Bl/6J compared to C57Bl/6N mice. Mol Metab. 2014;3(9):848–854. doi: 10.1016/j.molmet.2014.09.006 25506550 PMC4264561

[19] Fisher-Wellman KH, Ryan TE, Smith CD, Gilliam LA, Lin CT, Reese LR, et al. A Direct comparison of metabolic responses to high-fat diet in C57BL/6J and C57BL/6NJ Mice. Diabetes. 2016;65(11):3249–3261. doi: 10.2337/db16-0291 27495226 PMC5079634

[20] Williams JL, Paudyal A, Awad S, Nicholson J, Grzesik D, Botta J, et al. *Mylk3* null C57BL/6N mice develop cardiomyopathy, whereas *Nnt* null C57BL/6J mice do not. Life Sci Alliance. 2020;3(4):e201900593. doi: 10.26508/lsa.201900593 32213617 PMC7103425

[21] Wortmann M, Arshad M, Peters AS, Hakimi M, Böckler D, Dihlmann S. The C57Bl/6J mouse strain is more susceptible to angiotensin II-induced aortic aneurysm formation than C57Bl/6N. Atherosclerosis. 2021;318:8–13. doi: 10.1016/j.atherosclerosis.2020.11.032 33348068

[22] Vozenilek AE, Vetkoetter M, Green JM, Shen X, Traylor JG, Klein RL, et al. Absence of nicotinamide nucleotide transhydrogenase in C57BL/6J mice exacerbates experimental atherosclerosis. J Vasc Res. 2018;55(2):98–110. doi: 10.1159/000486337 29455203 PMC6287760

[23] Morales-Hernández A, Martinat A, Chabot A, Kang G, McKinney-Freeman S. Elevated oxidative stress impairs hematopoietic progenitor function in C57BL/6 substrains. Stem Cell Reports. 2018;11(2):334–347. doi: 10.1016/j.stemcr.2018.06.011 30017822 PMC6093083

[24] Usami M, Okada A, Taguchi K, Hamamoto S, Kohri K, Yasui T. Genetic differences in C57BL/6 mouse substrains affect kidney crystal deposition. Urolithiasis. 2018;46(6):515–522. doi: 10.1007/s00240-018-1040-3 29362828

[25] Toye AA, Lippiat JD, Proks P, Shimomura K, Bentley L, Hugill A, et al. A genetic and physiological study of impaired glucose homeostasis control in C57BL/6J mice. Diabetologia. 2005;48(4):675–686. doi: 10.1007/s00125-005-1680-z 15729571

[26] Ronchi JA, Figueira TR, Ravagnani FG, Oliveira HC, Vercesi AE, Castilho RF. A spontaneous mutation in the nicotinamide nucleotide transhydrogenase gene of C57BL/6J mice results in mitochondrial redox abnormalities. Free Radic Biol Med. 2013;63:446–456. doi: 10.1016/j.freeradbiomed.2013.05.049 23747984

[27] Matsuo N, Takao K, Nakanishi K, Yamasaki N, Tanda K, Miyakawa T. Behavioral profiles of three C57BL/6 substrains. Front Behav Neurosci. 2010;4:29. doi: 10.3389/fnbeh.2010.00029 20676234 PMC2912075

[28] Sankaran JS, Varshney M, Judex S. Differences in bone structure and unloading-induced bone loss between C57BL/6N and C57BL/6J mice. Mamm Genome. 2017;28(11–12):476–486. doi: 10.1007/s00335-017-9717-4 28913652

[29] Kahle M, Horsch M, Fridrich B, Seelig A, Schultheiß J, Leonhardt J, et al. Phenotypic comparison of common mouse strains developing high-fat diet-induced hepatosteatosis. Mol Metab. 2013;2(4):435–446. doi: 10.1016/j.molmet.2013.07.009 24327959 PMC3855089

[30] Kawashita E, Ishihara K, Nomoto M, Taniguchi M, Akiba S. A comparative analysis of hepatic pathological phenotypes in C57BL/6J and C57BL/6N mouse strains in non-alcoholic steatohepatitis models. Sci Rep. 2019;9(1):204. doi: 10.1038/s41598-018-36862-7 30659241 PMC6338790

[31] Rendina-Ruedy E, Hembree KD, Sasaki A, Davis MR, Lightfoot SA, Clarke SL, et al. A comparative study of the metabolic and skeletal response of C57BL/6J and C57BL/6N mice in a diet-induced model of type 2 diabetes. J Nutr Metab. 2015;2015:758080. doi: 10.1155/2015/758080 26146567 PMC4469802

[32] Sturm M, Becker A, Schroeder A, Bilkei-Gorzo A, Zimmer A. Effect of chronic corticosterone application on depression-like behavior in C57BL/6N and C57BL/6J mice. Genes Brain Behav. 2015;14(3):292–300. doi: 10.1111/gbb.12208 25752475

[33] Pohorec V, Križančić Bombek L, Skelin Klemen M, Dolenšek J, Stožer A. Glucose-stimulated calcium dynamics in beta cells from male C57BL/6J, C57BL/6N, and NMRI mice: a comparison of activation, activity, and deactivation properties in tissue slices. Front Endocrinol (Lausanne). 2022;13:867663. doi: 10.3389/fendo.2022.867663 35399951 PMC8988149

[34] Lusk G. ANIMAL CALORIMETRY Twenty-Fourth Paper. ANALYSIS OF THE OXIDATION OF MIXTURES OF CARBOHYDRATE AND FAT. J Biol Chem. 1924;59:41–42.

[35] Frayn KN. Calculation of substrate oxidation rates in vivo from gaseous exchange. J Appl Physiol Respir Environ Exerc Physiol. 1983;55(2):628–634. doi: 10.1152/jappl.1983.55.2.628 6618956

[36] Frühbeck G, Catalán V, Rodríguez A, Gómez-Ambrosi J. Adiponectin-leptin ratio: a promising index to estimate adipose tissue dysfunction. Relation with obesity-associated cardiometabolic risk. Adipocyte. 2018;7(1):57–62. doi: 10.1080/21623945.2017.1402151 29205099 PMC5915018

[37] Khisti RT, Wolstenholme J, Shelton KL, Miles MF. Characterization of the ethanol-deprivation effect in substrains of C57BL/6 mice. Alcohol. 2006;40(2):119–126. doi: 10.1016/j.alcohol.2006.12.003 17307648 PMC3082283

[38] Green ML, Singh AV, Zhang Y, Nemeth KA, Sulik KK, Knudsen TB. Reprogramming of genetic networks during initiation of the fetal alcohol syndrome. Dev Dyn. 2007;236(2):613–631. doi: 10.1002/dvdy.21048 17200951

[39] Mulligan MK, Ponomarev I, Boehm SL 2nd, Owen JA, Levin PS, Berman AE, et al. Alcohol trait and transcriptional genomic analysis of C57BL/6 substrains. Genes Brain Behav. 2008;7(6):677–689. doi: 10.1111/j.1601-183X.2008.00405.x 18397380

[40] Erikson CM, Douglas KT, Thuet TO, Richardson BD, Mohr C, Shiina H, et al. Independent of differences in taste, B6N mice consume less alcohol than genetically similar B6J mice, and exhibit opposite polarity modulation of tonic GABA_A_R currents by alcohol. Neuropharmacology. 2022;206:108934. doi: 10.1016/j.neuropharm.2021.108934 34933049 PMC9208337

[41] Ashworth A, Bardgett ME, Fowler J, Garber H, Griffith M, Curran CP. Comparison of neurological function in males and females from two substrains of C57BL/6 mice. Toxics. 2015;3(1):1–17. doi: 10.3390/toxics3010001 27081652 PMC4829364

[42] Bryant CD, Bagdas D, Goldberg LR, Khalefa T, Reed ER, Kirkpatrick SL, et al. C57BL/6 substrain differences in inflammatory and neuropathic nociception and genetic mapping of a major quantitative trait locus underlying acute thermal nociception. Mol Pain. 2019;15: 1744806918825046. doi: 10.1177/1744806918825046 30632432 PMC6365993

[43] Bryant CD, Zhang NN, Sokoloff G, Fanselow MS, Ennes HS, Palmer AA, et al. Behavioral differences among C57BL/6 substrains: implications for transgenic and knockout studies. J Neurogenet. 2008;22(4):315–331. doi: 10.1080/01677060802357388 19085272 PMC3697827

[44] Akinola LS, McKiver B, Toma W, Zhu AZX, Tyndale RF, Kumar V, et al. C57BL/6 substrain differences in pharmacological effects after acute and repeated nicotine administration. Brain Sci. 2019;9(10):244. doi: 10.3390/brainsci9100244 31546627 PMC6827359

[45] Bothe GW, Bolivar VJ, Vedder MJ, Geistfeld JG. Genetic and behavioral differences among five inbred mouse strains commonly used in the production of transgenic and knockout mice. Genes Brain Behav. 2004;3(3):149–157. doi: 10.1111/j.1601-183x.2004.00064.x 15140010

[46] Karthivashan G, Park SY, Kim JS, Cho DY, Ganesan P, Choi DK. Comparative studies on behavioral, cognitive and biomolecular profiling of ICR, C57BL/6 and its sub-strains suitable for scopolamine-induced amnesic models. Int J Mol Sci. 2017;18(8):1735. doi: 10.3390/ijms18081735 28792471 PMC5578125

[47] Cardin S, Scott-Boyer MP, Praktiknjo S, Jeidane S, Picard S, Reudelhuber TL, et al. Differences in cell-type-specific responses to angiotensin II explain cardiac remodeling differences in C57BL/6 mouse substrains. Hypertension. 2014;64(5):1040–1046. doi: 10.1161/HYPERTENSIONAHA.114.04067 25069667

[48] Duan L, Davis JS, Woolbright BL, Du K, Cahkraborty M, Weemhoff J, et al. Differential susceptibility to acetaminophen-induced liver injury in sub-strains of C57BL/6 mice: 6N versus 6J. Food Chem Toxicol. 2016;98(Pt B):107–118. doi: 10.1016/j.fct.2016.10.021 27773698 PMC5123947

[49] Bufi R, Korstanje R. The impact of genetic background on mouse models of kidney disease. Kidney Int. 2022. doi: 10.1016/j.kint.2022.03.020 35429495 PMC9233094

[50] Ma Q, Grigorescu M, Schreiber A, Kettritz R, Lindenmeyer M, Anders HJ, et al. Genetic background but not intestinal microbiota after co-housing determines hyperoxaluria-related nephrocalcinosis in common inbred mouse strains. Front Immunol. 2021;12:673423. doi: 10.3389/fimmu.2021.673423 33968083 PMC8100042

[51] Kang SK, Hawkins NA, Kearney JA. C57BL/6J and C57BL/6N substrains differentially influence phenotype severity in the *Scn1a* ^*+/-*^ mouse model of Dravet syndrome. Epilepsia Open. 2019;4(1):164–169. doi: 10.1002/epi4.12287 30868126 PMC6398090

[52] Williams JL, Hall CL, Meimaridou E, Metherell LA. Loss of Nnt increases expression of oxidative phosphorylation complexes in C57BL/6J hearts. Int J Mol Sci. 2021;22(11):6101. doi: 10.3390/ijms22116101 34198873 PMC8201288

[53] Ulmasov B, Oshima K, Rodriguez MG, Cox RD, Neuschwander-Tetri BA. Differences in the degree of cerulein-induced chronic pancreatitis in C57BL/6 mouse substrains lead to new insights in identification of potential risk factors in the development of chronic pancreatitis. Am J Pathol. 2013;183(3):692–708. doi: 10.1016/j.ajpath.2013.05.020 23845568 PMC3763767

[54] Watkins-Chow DE, Pavan WJ. Genomic copy number and expression variation within the C57BL/6J inbred mouse strain. Genome Res. 2008;18(1):60–66. doi: 10.1101/gr.6927808 18032724 PMC2134784

[55] Timmermans S, Van Montagu M, Libert C. Complete overview of protein-inactivating sequence variations in 36 sequenced mouse inbred strains. Proc Natl Acad Sci U S A. 2017;114(34):9158–9163. doi: 10.1073/pnas.1706168114 28784771 PMC5576813

[56] Flynn JM, Brown EJ, Clark AG. Copy number evolution in simple and complex tandem repeats across the C57BL/6 and C57BL/10 inbred mouse lines. G3 (Bethesda). 2021;11(8):jkab184. doi: 10.1093/g3journal/jkab184 34849804 PMC8496272

[57] Pivovarova O, Höhn A, Grune T, Pfeiffer AF, Rudovich N. Insulin-degrading enzyme: New therapeutic target for diabetes and Alzheimer’s disease? Ann Med. 2016;48(8):614–624. doi: 10.1080/07853890.2016.1197416 27320287

[58] Ravussin A, Youm YH, Sander J, Ryu S, Nguyen K, Varela L, et al. Loss of nucleobindin-2 causes insulin resistance in obesity without impacting satiety or adiposity. Cell Rep. 2018;24(5):1085–1092.e6. doi: 10.1016/j.celrep.2018.06.112 30067966 PMC6223120

[59] The Jackson Laboratory. Mice strain B6.FVB-Tg(EIIa-cre)C5379Lmgd/J. https://www.jax.org/strain/003724.

[60] Rawle DJ, Le TT, Dumenil T, Bishop C, Yan K, Nakayama E, et al. Widespread discrepancy in *Nnt* genotypes and genetic backgrounds complicates granzyme A and other knockout mouse studies. elife. 2022;11:e70207. doi: 10.7554/eLife.70207 35119362 PMC8816380

