# Supplementary figures and images for: Metabolic differences and differentially expressed genes between C57BL/6J and C57BL/6N mice substrains
Source: PLoS One. 2022 Dec 22;17(12):e0271651. doi: 10.1371/journal.pone.0271651 (PMC9778930; doi:10.1371/journal.pone.0271651)

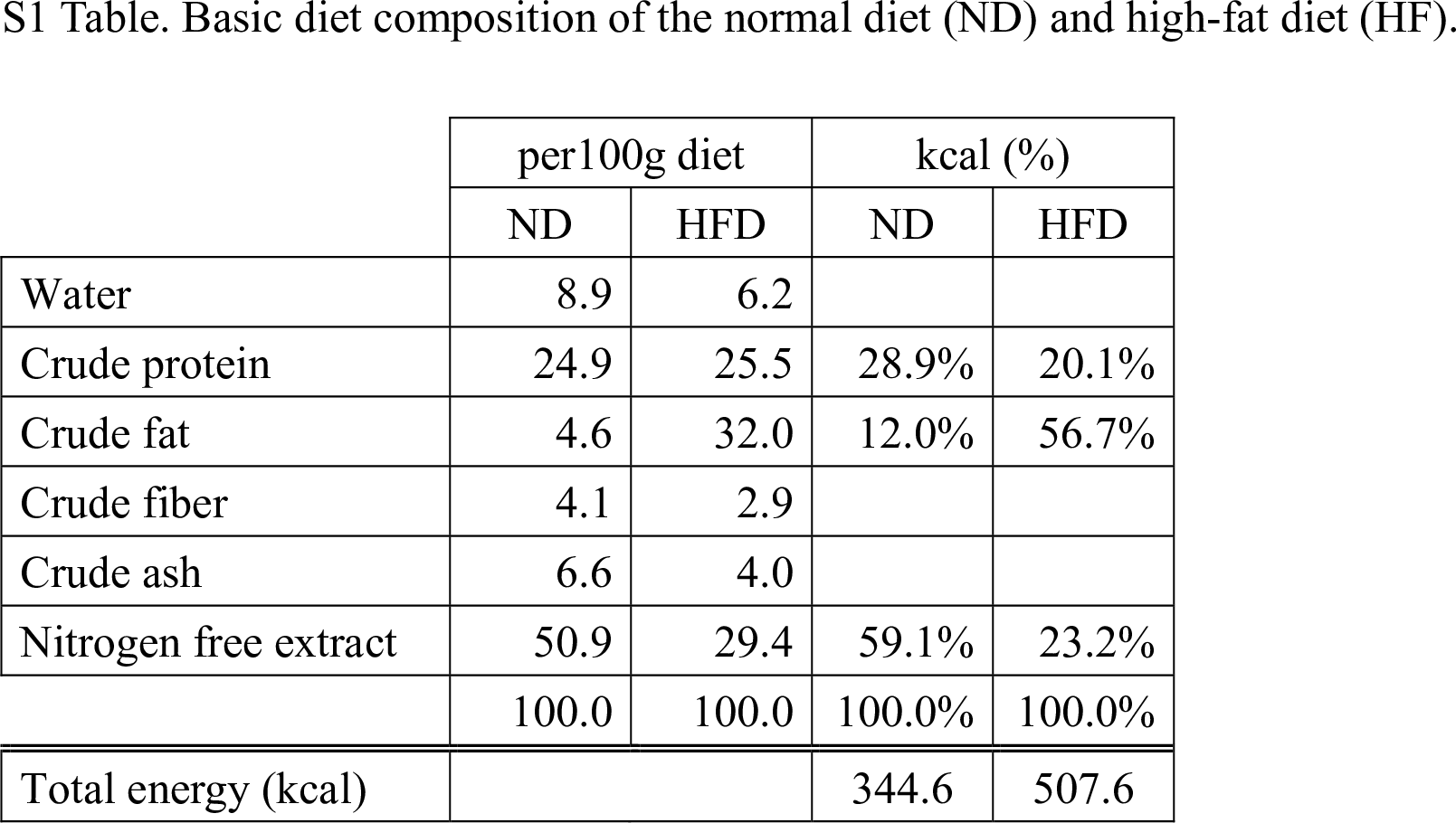

Supplement: S1 Table — (TIF) [file pone.0271651.s001.tif]

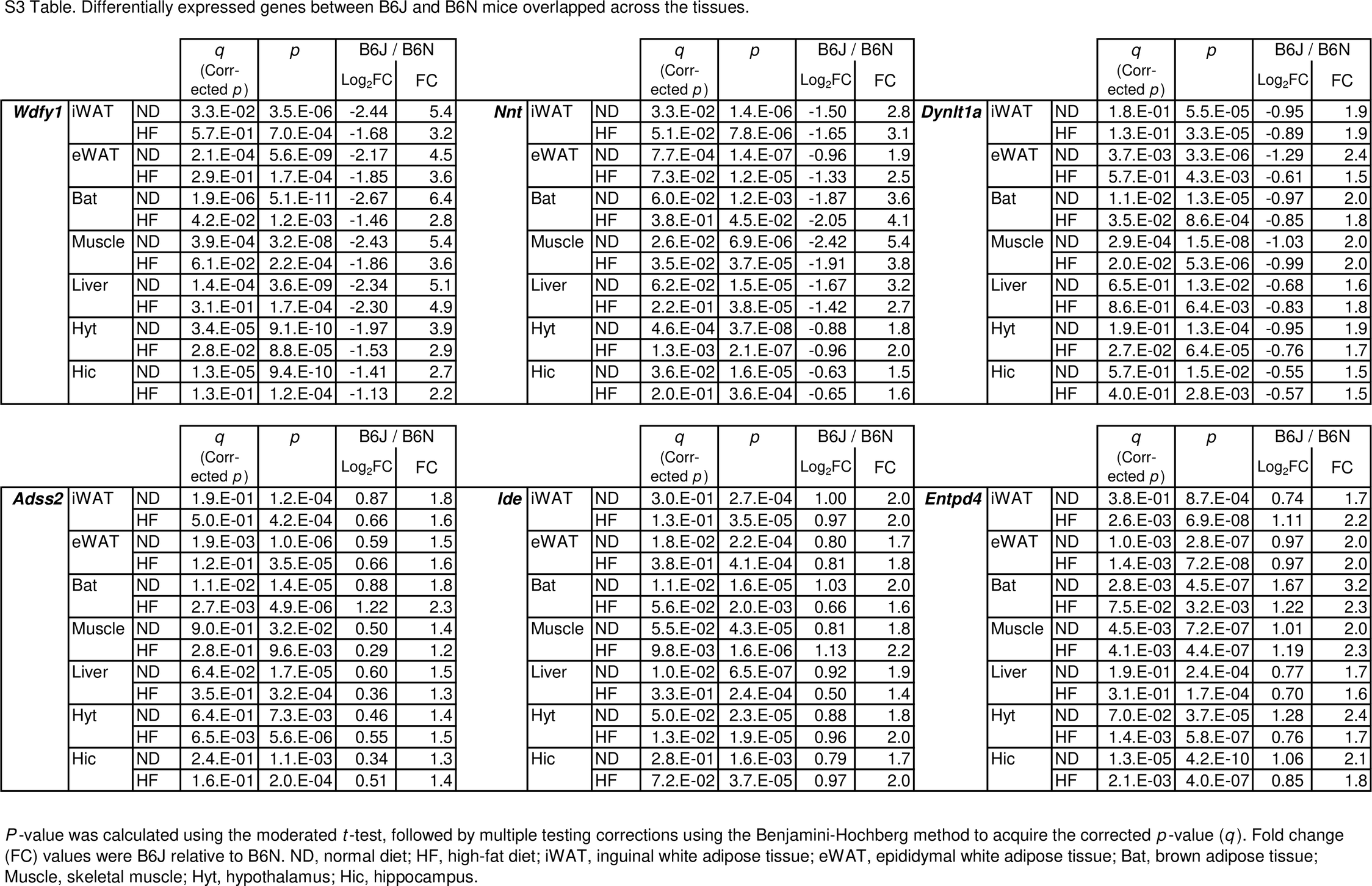

Supplement: S3 Table — P-values were calculated using the moderated t-test, followed by multiple testing corrections using the Benjamini-Hochberg method to acquire the corrected p-value (q). Fold change (FC) values were B6J relative to B6N. ND, normal diet; HF, high-fat diet; iWAT, inguinal white adipose tissue; eWAT, epididymal white adipose tissue; Bat, brown adipose tissue; Muscle, skeletal muscle; Hyt, hypothalamus; Hic, hippocampus. (TIF) [file pone.0271651.s003.tif]

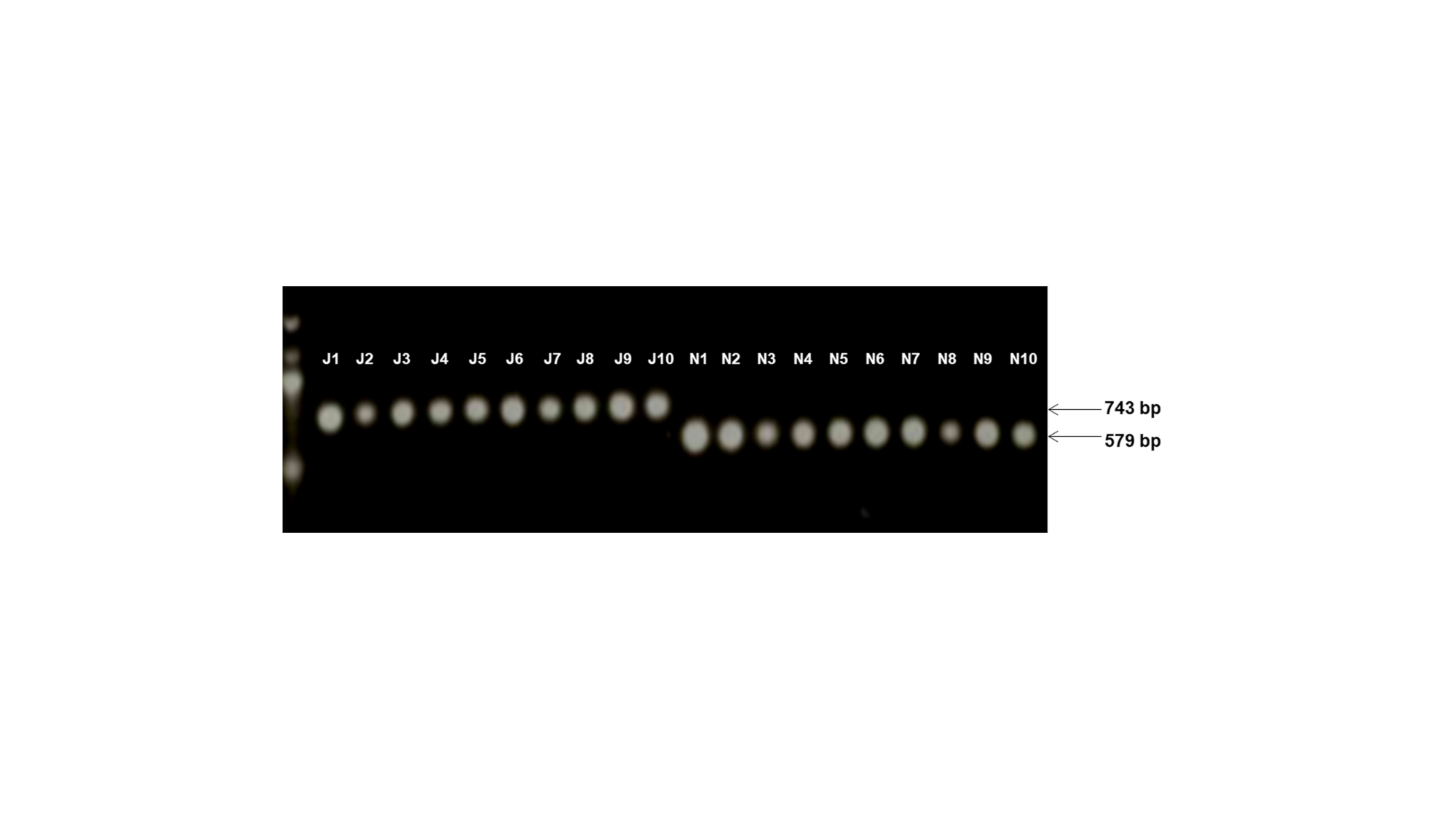

Supplement: S1 Fig — Polymerase chain reaction analysis of Nnt alleles. DNA was obtained from the tail of B6J and B6N mice. The amplification products were 579 bp and 743 bp for the wild-type and mutant alleles, respectively. (TIF) [file pone.0271651.s004.tif]

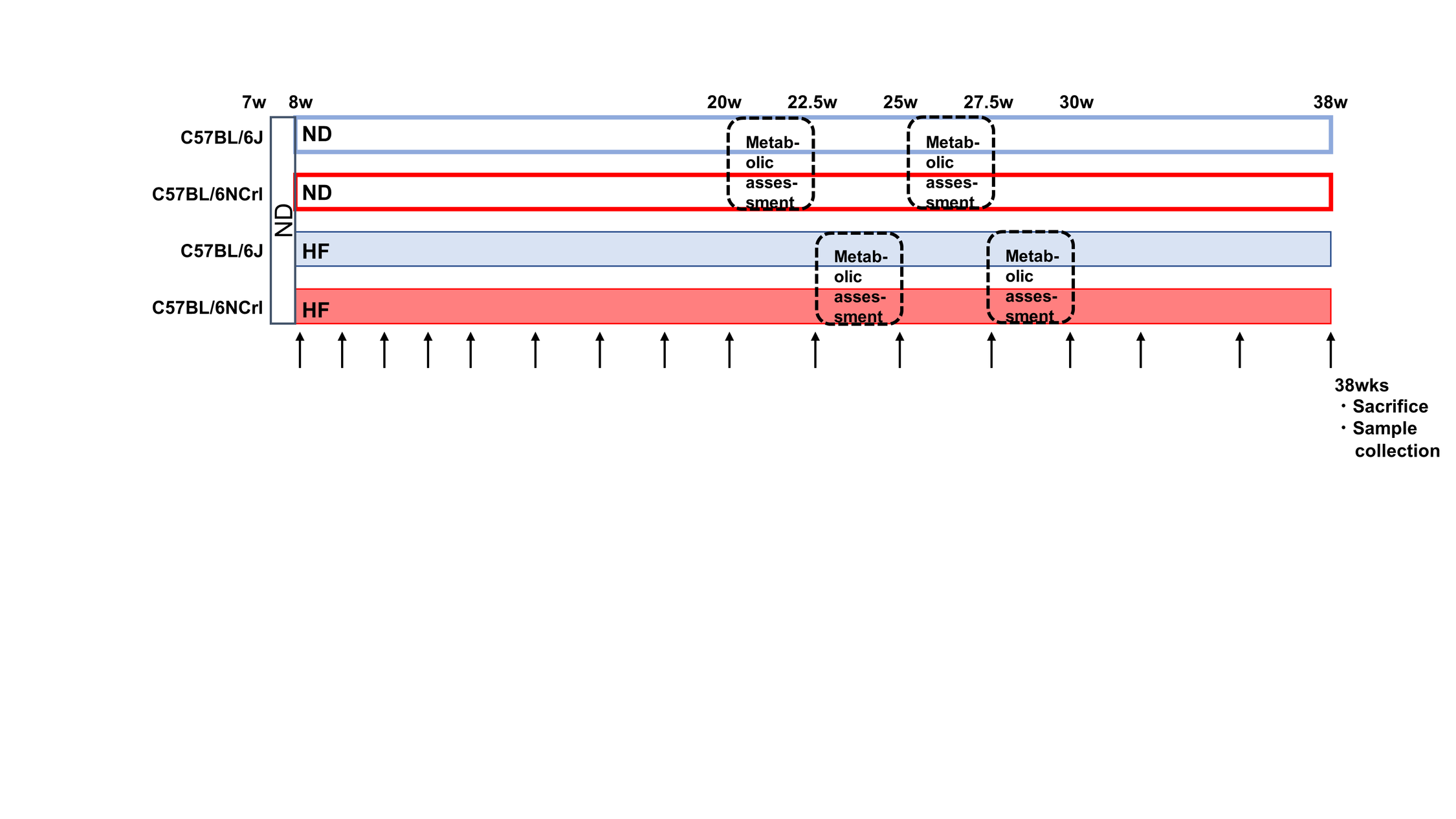

Supplement: S2 Fig — Four groups of animals were used. Colored boxes represent mouse substrain and diet groups; open blue box, normal diet (ND)-fed C57BL/6J (B6J); open red box, ND-fed C57BL/6NCrl (B6N); filled blue box, high-fat diet (HF)-fed B6J; filled red box, HF-fed B6N. Body weight was measured at the ages indicated by arrows. Mice were subjected to an indirect calorimetry system at the time points indicated as “metabolic assessment”. All animals were sacrificed and samples were taken at 38 weeks of age. (TIF) [file pone.0271651.s005.tif]

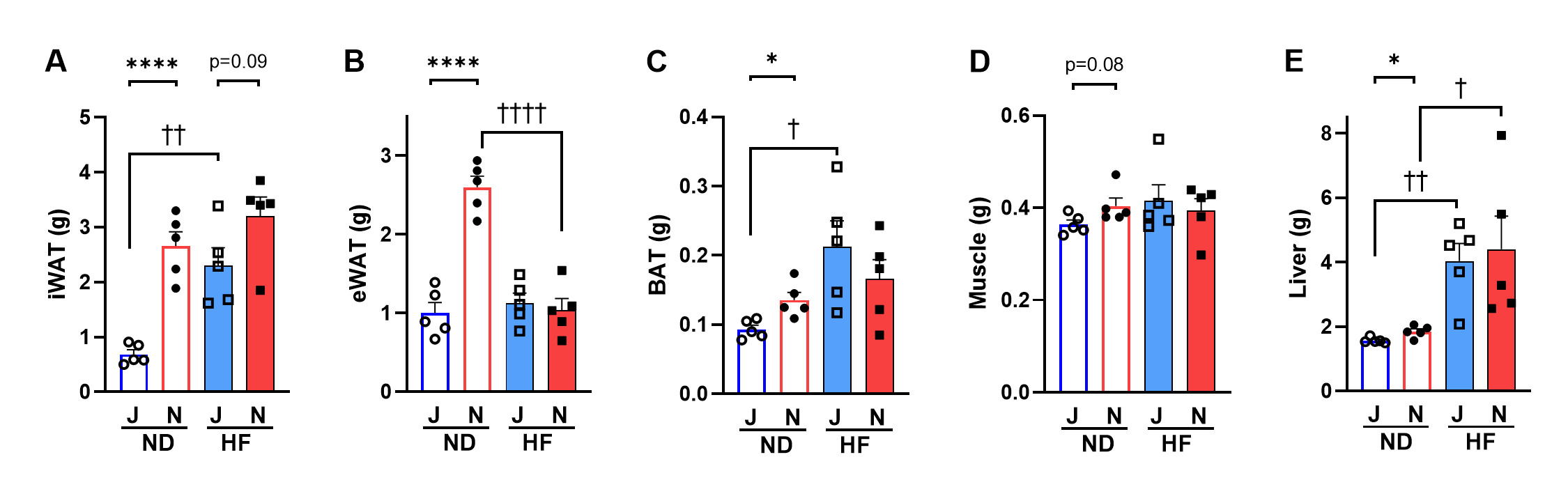

Supplement: S3 Fig — (A) iWAT, inguinal white adipose tissue; (B) eWAT, epididymal white adipose tissue; (C) BAT, brown adipose tissue; (D) Muscle, skeletal muscle; (E) Liver. Bars are representative of means + SEM (n = 5) for the B6J (blue color) and B6N (red color) groups. Two-way ANOVA indicated a significant interaction between substrain and diet for eWAT (p<0.0001; F = 38.56; Df = 16). There was a significant effect of strain on iWAT (p<0.0001; F = 27.49; Df = 16) independent of diet, and significant effect of diet on iWAT (p<0.01; F = 15.61; Df = 16), BAT (p <0.01; F = 9.50; Df = 16) and liver (p<0.001; F = 18.48; Df = 16) independent of strain. Individual means were compared within groups by unpaired Student’s t-test. Asterisks (*) and daggers (†) indicate significant differences between B6J and B6N substrain groups, and ND and HF diet groups, respectively (** p<0.01 and **** p<0.0001, † p<0.05, †† p<0.01, and †††† p<0.0001). J, B6J substrain; N, B6N substrain; ND, normal diet; HF, high-fat diet; open circles, ND-fed B6J; filled circles, ND-fed B6N; open squares, HF-fed B6J; filled squares, HF-fed B6N. (TIF) [file pone.0271651.s006.tif]
